# Repeat Interruptions Modify Age at Onset in Myotonic Dystrophy Type 1 by Stabilizing *DMPK* Expansions in Somatic Cells

**DOI:** 10.3389/fgene.2018.00601

**Published:** 2018-11-27

**Authors:** Jovan Pešović, Stojan Perić, Miloš Brkušanin, Goran Brajušković, Vidosava Rakočević-Stojanović, Dušanka Savić-Pavićević

**Affiliations:** ^1^Center for Human Molecular Genetics, Faculty of Biology, University of Belgrade, Belgrade, Serbia; ^2^School of Medicine, University of Belgrade, Belgrade, Serbia; ^3^Neurology Clinic, Clinical Center of Serbia, Belgrade, Serbia

**Keywords:** age at onset, CTG expansion, *DMPK*, myotonic dystrophy 1, repeat interruptions, somatic instability

## Abstract

CTG expansions in *DMPK* gene, causing myotonic dystrophy type 1 (DM1), are characterized by pronounced somatic instability. A large proportion of variability of somatic instability is explained by expansion size and patient’s age at sampling, while individual-specific differences are attributed to additional factors. The age at onset is extremely variable in DM1, and inversely correlates with the expansion size and individual-specific differences in somatic instability. Three to five percent of DM1 patients carry repeat interruptions and some appear with later age at onset than expected for corresponding expansion size. Herein, we characterized somatic instability of interrupted *DMPK* expansions and the effect on age at onset in our previously described patients. Repeat-primed PCR showed stable structures of different types and patterns of repeat interruptions in blood cells over time and buccal cells. Single-molecule small-pool PCR quantification of somatic instability and mathematical modeling showed that interrupted expansions were characterized by lower level of somatic instability accompanied by slower progression over time. Mathematical modeling demonstrated that individual-specific differences in somatic instability had greater influence on age at onset in patients with interrupted expansions. Therefore, repeat interruptions have clinical importance for disease course in DM1 patients due to stabilizing effect on *DMPK* expansions in somatic cells.

## Introduction

Myotonic dystrophy type 1 (DM1, MIM #160900) is a multisystem disease that affects skeletal muscles, heart, brain, eyes and endocrine system, and has an extremely variable age of disease onset (Harper, 2001). It is an autosomal dominant disorder caused by an expansion of 50 to a few thousands repeats in (CTG)_n_ tract located at the 3′ untranslated region of the *DMPK* gene (MIM ^∗^605377) (Brook et al., 1992; Fu et al., 1992; Mahadevan et al., 1992). The number of CTG repeats broadly correlates with the disease severity (Harley et al., 1993; De Antonio et al., 2016).

Although CTG repeat tract at the *DMPK* locus was considered to be uninterrupted in both wild-type and expanded alleles, less than a decade ago expanded alleles containing repeat interruptions (also known as variant repeats) were identified (Musova et al., 2009; Braida et al., 2010). Since then, CCG, GGC, CTC and CAG interruptions have been described within 3’ and 5’ ends of *DMPK* expansions, with various patterns of CCG repeats being the most commonly detected (Musova et al., 2009; Braida et al., 2010; Santoro et al., 2013; Botta et al., 2017; Pešović et al., 2017; Cumming et al., 2018; Tomé et al., 2018). The frequency of repeat interruptions has been estimated at 3–5% in DM1 patients of different origin (Musova et al., 2009; Braida et al., 2010; Santoro et al., 2013; Botta et al., 2017; Pešović et al., 2017; Cumming et al., 2018). Even though the majority of clinical signs in those patients were associated with DM1 phenotype, some patients appeared with milder or atypical symptoms (Musova et al., 2009; Pešović et al., 2017; Cumming et al., 2018), even atypical neurological phenotype (Braida et al., 2010), and without described congenital form. A noticeable feature of some DM1 patients with repeat interruptions described by us (Pešović et al., 2017) and others (Musova et al., 2009; Botta et al., 2017) was a later age at onset than expected for the corresponding number of CTG repeats. These findings have made repeat interruptions a potential genetic modifier of DM1 phenotype, likewise described in spinocerebellar ataxia type 10 (Matsuura et al., 2006).

Repeat interruptions are known *cis*-acting modifiers with a stabilizing effect either on wild type (Cleary and Pearson, 2003) or expanded (Moseley et al., 2000; Matsuura et al., 2006; Gao et al., 2008; Hu et al., 2017) repeat arrays associated with numerous unstable repeat expansion diseases. Therefore, one of the mechanisms by which repeat interruptions can influence the effect of DM1 causing mutation is genetic stabilization of CTG repeat array.

Accumulated data have indicated that interrupted *DMPK* expansions were prone to relatively stable intergenerational transmissions or even contractions in almost all described cases (Musova et al., 2009; Braida et al., 2010; Botta et al., 2017; Pešović et al., 2017; Tomé et al., 2018). Contrary to transmissions of uninterrupted *DMPK* expansions (Lavedan et al., 1993; Rakocević-Stojanović et al., 2005), these transmissions were independent on the sex of the transmitting parent (Pešović et al., 2017) and likely explain the absence of congenital cases (Braida et al., 2010; Tomé et al., 2018).

A pronounced instability of uninterrupted *DMPK* expansions in somatic cells leads to somatic mosaicism that is tissue-specific, age-dependent, biased toward further expansions throughout the patient’s life (Monckton et al., 1995; Wong et al., 1995; Martorell et al., 1998) and contributes to progressive nature of the symptoms (Morales et al., 2012). In a small number of DM1 patients studied so far, a reduced instability of interrupted *DMPK* expansions in blood cells was described (Braida et al., 2010; Cumming et al., 2018; Tomé et al., 2018). In addition, it has been suggested that a low level of somatic instability may partially explain the mild symptoms seen in some of these patients (Cumming et al., 2018).

Quantification of somatic instability by small-pool PCR in a large group of DM1 patients with uninterrupted expansions and linear regression modeling have shown that a large proportion of variability of somatic instability is explained by the progenitor allele length and the patient’s age at sampling (Morales et al., 2012). The unexplained, inter-individual variability is a heritable quantitative trait influenced by individual-specific genetic and environmental factors (Morales et al., 2012). The same study has also shown that age at onset inversely correlates with the progenitor allele length and inter-individual variability of somatic instability. Therefore, identification of factors that contribute to individual-specific differences in the level of somatic instability may help to gain a better understanding of factors influencing variability of age at onset in DM1 patients.

Given that some of our previously genetically and clinically characterized DM1 patients with repeat interruptions appeared to have later age at onset than expected for the corresponding expansion size (Pešović et al., 2017), herein we characterized mutational dynamics of interrupted *DMPK* expansions in somatic cells and examined, for the first time, whether repeat interruptions modified age at onset by influencing the level of somatic instability.

## Materials and Methods

### Subjects

The study included seven DM1 patients with interrupted *DMPK* expansions from Serbia, who gave informed consent. Six patients (DF1-1, DF1-2, DF1-3, DF2-1, DF3-1, and DF5-2) from four families have been previously genetically characterized (Pešović et al., 2017) (Table 1). Patient DF3-2 has been recently identified with the pattern of repeat interruptions identical to her mother (DF3-1) (Table 1) and an intergenerational transmission accompanied by an apparent contraction. Patients underwent a detailed clinical examination at the Neurology Clinic, Clinical Center of Serbia, Belgrade at two different time points (except DF3-2) in the period between 2013 and 2017. The first clinical examination was carried out upon the initial detection of interrupted *DMPK* expansions, and the second one approximately two and half to four years later. Clinical characteristics of patients at the first examination have been described (Pešović et al., 2017), and no significant progression of the disease was observed at the second examination. Blood samples were collected at both time points, while the buccal swab samples were obtained from five patients at their second examination (DF1-1, DF1-2, DF1-3, DF5-2, and DF5-3). Four DM1 patients with uninterrupted *DMPK* expansions served as controls (herein referred to as the control group) (Table 1). One of them was a sister (DF5-3) of the patient with a single, *de novo* CTC repeat interruption (DF5-2). The remaining three were unrelated to each other and to patients carrying repeat interruptions, and their blood DNA samples were available from two different time points ranging the time interval of 8–11 years. These DNA samples belong to the DNA bank that accompanies the Serbian patient registry for myotonic dystrophies. The study was approved by the Ethical Committee of the University of Belgrade, School of Medicine (number 339/5). It was performed in accordance with the ethical standards of the 1964 Declaration of Helsinki and its later amendments.

**Table 1:** Main features of DM1 patients analyzed in this study.

Patient ID	Sex	Interruption pattern	AO	Onset symptoms	PAL	AS t1	10th t1	AS t2	10th t2
DF1-1	F	(CTG)_n_(**CCG**CTG)_3_(CTG)_4_(**CCG**CTG)_2_CTG**CCG**(CTG)_17_	39.5	Hand grip myotonia	520	61.5	459	65.5	443
DF1-2	M		30.5	Hand grip myotonia	350	34.5	348	38.5	384
DF1-3	M		15.5	Hand grip myotonia	450	35.5	441	39.5	469
DF2-1	M	(CTG)_n_(**CCG**)_36_(CTG)_n_**CCG**(CTG)_7_**CCG**(CTG)_12_	40.5	Proximal arm and leg weakness	320	45.5	371	48	350
DF3-1	F	(CTG)_n_(**CCG**)_3_(CTG)_6_(**CCG**)_3_(CTG)_7_**CCG**(CTG)_8_**CCG**(CTG)_8_	45.5	Proximal leg weakness	240	50	266.5	52.5	289
DF3-2	F		31.5	Hand grip myotonia, leg pain	NA	NA	NA	31.5	187
DF5-2	F	(CTG)_n_**CTC**(CTG)_26_	22.5	Masticatory muscles myotonia	250	26.5	235	30.5	262
DF5-3^∗^	F	pure CTG expansion	21.5	Hand grip myotonia	300	23.5	314	27.5	422
MD70^∗^	F		35.5	Hand grip myotonia	NA	50.5	106	61.5	146
MD179^∗^	F		13.5	Hand grip myotonia	NA	14.5	326	23	415
MD180^∗^	F		14.5	Hand grip myotonia	NA	22.5	400	30.5	462


*AO, age at onset, self-reported by the patients in years; PAL, the progenitor allele length in the number of repeats; AS, age at sampling in years; 10th, the 10th percentile allele length in the number of repeats; t1 and t2, the first and the second sampling time point, respectively; ^∗^, analyzed patients with uninterrupted *DMPK* expansions; NA, not available. Data for interruption pattern and PAL are from our previous study (Pešović et al., 2017). PAL was estimated at t1 as a sharp lower boundary of expansion range (Monckton et al., 1995) using small-pool PCR with ∼300 pg of input DNA. In this study, patients were analyzed by single-molecule small pool PCR, and we used the 10th percentile allele length as an unbiased measurement (Higham, 2013) of the progenitor allele length.*

### Molecular Genetic Analyses

Genomic DNA was extracted from blood and buccal swab samples using QIAamp^®^ DNA Blood Mini Kit (Qiagen, Hilden, Germany). To determine the patterns of repeat interruptions in buccal cells and to investigate them in blood cells over time, repeat-primed PCR was performed as previously described (Pešović et al., 2017). Additional primer P5CCG (5′-AGCGTCTACTGTCTCGGCACTTGCCCGCCGCCGCCG-3′) was used in repeat-primed PCR to determine the number of repeats in a large CCG block in DF2-1 patient.

Quantification of somatic instability of *DMPK* expanded alleles was performed by single-molecule small-pool PCR containing ∼30–60 pg of DNA (5–10 genomic equivalents). PCR protocol was as previously described (Savić et al., 2002), with the exception of DF2-1, for whom a protocol developed for the detection of *FMR1* CGG expansions (Saluto et al., 2005) was used with primers 101 and 102 (Brook et al., 1992). Briefly, upon transfer to membrane, products were hybridized with (CAG)_12_ oligonucleotide labeled at the 3′ end with DIGddUTP, and visualized by chemiluminescence detection using anti-DIG alkaline phosphatase and CDP-star (Roche Life Science, Mannheim, Germany). Single-molecule small-pool PCR products were sized using Core Laboratory Image Quantification Software – CLIQS (TotalLab Ltd., Newcastle upon Tyne, United Kingdom). At least 200 alleles per sample were amplified and sized, and used to obtain allele frequency distribution. The 10th percentile of allele frequency distribution was used as an estimate for the progenitor allele length (Higham, 2013).

### Statistical Analyses

To examine tissue specificity of somatic mosaicism, the allele frequency distributions in blood and buccal cells were compared in each patient by Wilcoxon–Mann–Whitney test, after testing data normality using Shapiro–Wilk test.

To characterize mutational dynamics of *DMPK* expansions in blood cells, the observed and expected levels of somatic instability were compared in each group of patients using Wilcoxon signed-rank test. The difference in the number of repeats at the 10th and the 90th percentile of the allele frequency distribution was used as a measure of observed level of somatic instability (Morales et al., 2012; Higham, 2013). To estimate the expected level of somatic instability, the linear regression models that correlate somatic instability with the progenitor allele length and age at sampling were considered (Morales et al., 2012). The linear regression models were retested using the 10th percentile allele length of 136 patients carrying uninterrupted *DMPK* expansions (herein referred to as the reference group) (Morales et al., 2012) and patients analyzed in this study. Retesting was performed separately for the first and the second sampling time point (Supplementary Table 1). Model 8^∗^ explained 90% of variability of somatic instability at both time points, and was used to estimate the expected level of somatic instability.

To examine inter-individual variability of somatic instability according to model 8^∗^, residual variance was considered. This residual variance reflects individual-specific differences in the level of somatic instability that is not accounted for by the progenitor allele length and age at sampling. It is attributed to individual-specific factors (genetic and environmental). Residuals were standardized to a mean of zero and a standard deviation (SD) of 1. Standardized residuals were compared between patients analyzed in this study and the reference group by Wilcoxon–Mann–Whitney test, after testing data normality using the Shapiro–Wilk test.

To characterize mutational dynamics of somatic instability in blood cells over time, the allele frequency distributions from two time points were compared in each patient by Wilcoxon–Mann–Whitney test. In addition, the observed and expected expansion size increments over time were compared in each group of patients using Wilcoxon signed-rank test. The expected expansion size increment over time was estimated using the linear regression model explaining ∼56% of variability by the modal expansion size and time interval between samplings as independent variables (Martorell et al., 1998). As a large number of alleles sized by single-molecule small-pool PCR resulted in non-normal allele frequency distributions, the mode allele length was assumed as corresponding measure of the modal expansion size. In the original study, the modal expansion size was determined at the point of highest band intensity on the autoradiograph of genomic Southern blot or at the center of the smear for very diffuse bands (Martorell et al., 1998). The observed expansion size increment over time was calculated as difference between the mode allele length at the first and the second time point.

To characterize mutational dynamics of *DMPK* expansions without interrupted part (herein referred to as the 5′ end) in blood cells over time, we used the same approach as above described for the whole run of repeats. The size of the 5′ ends was obtained by subtracting the corresponding interrupted part at the 3′ end from all detected expanded alleles in each sample. Retested model 8^∗∗^ (Morales et al., 2012) for both sampling time points explained 90% of variability of somatic instability (Supplementary Table 2).

To analyze age at onset, the observed and expected age at onset were compared in each group of patients by Wilcoxon signed-rank test. The observed age at onset was self-reported by the patients (Table 1). To estimate the expected age at onset, the following linear regression models were considered (Morales et al., 2012): models 9 and 10, correlating age at onset with the progenitor allele length, and model 11, correlating age at onset with the progenitor allele length and residual variance of somatic instability obtained from model 8^∗^. The models were retested using the 10th percentile allele length of 121 symptomatic patients from the reference group (Morales et al., 2012) and patients examined in this study. Retesting was again performed separately for the first and the second sampling time point. Retested models explained ∼43% (model 9^∗^), ∼49% (model 10^∗^) and ∼55% (model 11^∗^) of variability of age at onset (Supplementary Table 3). Models 10^∗^ and 11^∗^ were used to assess the expected age at onset.

To examine inter-individual variability of age at onset according to models 10^∗^ and 11^∗^, standardized residuals were compared between patients analyzed in this study and the reference group using Wilcoxon–Mann–Whitney test, after testing data normality using the Shapiro–Wilk test. Residual variance in model 10^∗^ reflects individual-specific differences in age at onset that are not explained by the progenitor allele length. Residual variance in model 11^∗^ reflects individual-specific differences in age at onset that are not explained by the progenitor allele length and by inter-individual variability of somatic instability according to model 8^∗^. To assess contribution of inter-individual variability of somatic instability, standardized residuals from model 11^∗^ were compared with standardized residuals from model 10^∗^ in each group of patients using Wilcoxon signed-rank test.

Statistical analyses were performed in the R environment for statistical computing and visualization ver 3.3.3 (R Core Team, 2016). Statistical analyses were performed for the first and the second sampling time point, where appropriate. The significance level was set at 0.05 (two-tailed *p*-value) in all tests.

## Results

### Interrupted *DMPK* Expansions Show Tissue-Specific Somatic Instability Prone to Further Expansions

**FIGURE 1:**
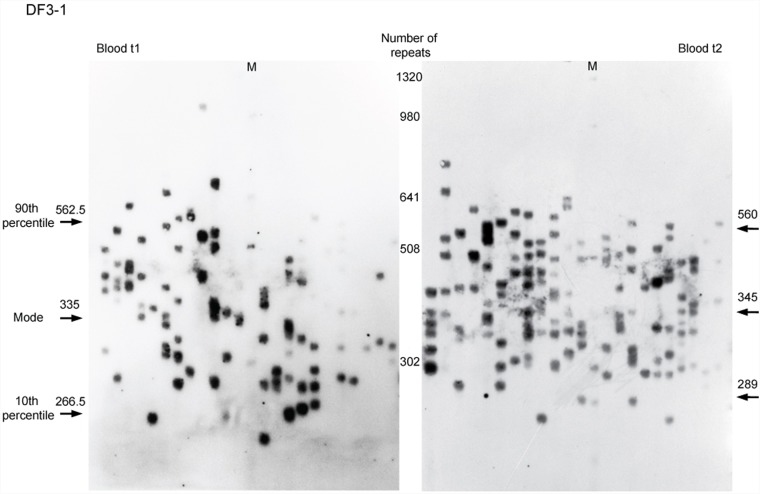
Single-molecule small-pool PCR analysis of *DMPK* expanded alleles with repeat interruptions. Analysis of the blood sample for DF3-1 when she was 50 years old (the first time point, blood t1) and 52.5 years old (the second time point, blood t2), showing allele size heterogeneity. Each lane of agarose gel (24 cm in length) contained PCR products amplified from ∼30–60 pg of DNA (5–10 genomic equivalents). Arrows with number indicate the position and size of the mode, the 10th and the 90th percentile allele length in number of trinucleotide repeats, inferred from more than 200 alleles sized per sample. M, DNA Molecular Weight Marker X (Roche Life Science, Mannheim, Germany) shown in number of CTG repeats.

A total number of 5,701 alleles were amplified and sized by single-molecule small-pool PCR, with at least 200 alleles per sample (the dataset is given in Supplementary Table 4). 4,572 alleles were amplified from blood sampled at two time points from six DM1 patients carrying interrupted expansions and four patients with uninterrupted expansions, and at one time point from DF3-2, carrying an interrupted expansion. Additional 1,129 alleles were amplified from buccal swab samples, available from four patients with repeat interruptions and one with pure CTG repeats. A representative blot of a single-molecule small-pool PCR is shown in Figure 1.

**FIGURE 2:**
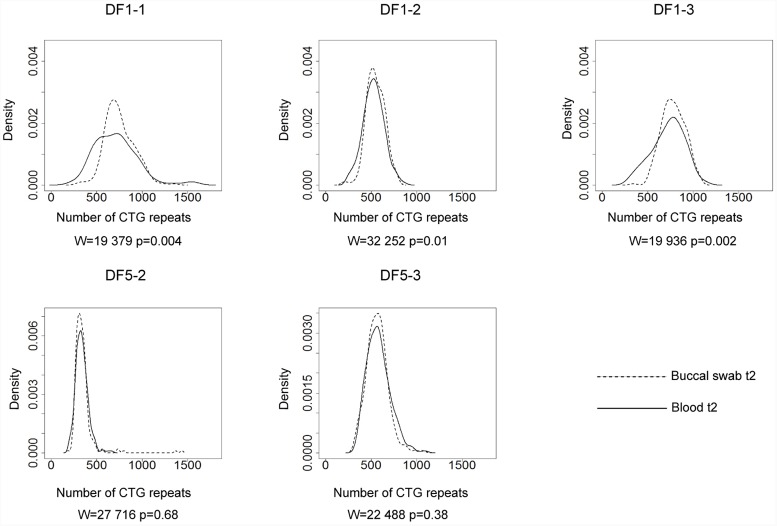
Tissue specific somatic instability of *DMPK* expansions. Density plots show the allele frequency distributions skewed toward larger expansions in blood (solid lines) and buccal cells (dashed lines), sampled at the same time point (t2). All allele frequency distributions were derived from sizing at least 200 alleles and their comparisons were performed by Wilcoxon–Mann–Whitney test (*W* and *p*-values are shown). DF1 family members **(top graphs)**, carrying identical pattern of CGG interruptions, showed difference in the allele frequency distribution between examined tissues. DF5 family members **(bottom graphs)** showed no difference in the allele frequency distribution between examined tissues. DF5-2 has a single *de novo* CTC interruption, while her sister DF5-3 has an uninterrupted *DMPK* expansion.

Allele frequency distribution in all samples was skewed toward larger alleles. Comparison of the allele frequency distribution between blood and buccal cells showed statistical difference in DF1 family members carrying runs of CCGCTG hexamer (mother DF1-1, *W* = 19,379, *p* = 0.004; son DF1-2, *W* = 32,252, p = 0.01; son DF1-3, *W* = 19,936, *p* = 0.002) (Figure 2). The progenitor allele length was higher (50–150 repeats more) and the level of somatic instability was lower in buccal than in blood cells (Figure 2 and Supplementary Table 5). Patient DF5-2, carrying a single *de novo* CTC interruption, showed no statistical difference in the allele frequency distribution between examined tissues (*W* = 27,716, *p* = 0.68) (Figure 2 and Supplementary Table 5). The similar result was obtained for her sister (DF5-3) carrying an uninterrupted *DMPK* expansion (*W* = 22,488, *p* = 0.38) (Figure 2 and Supplementary Table 5). Therefore, *DMPK* expansions with repeat interruptions were characterized by allele length heterogeneity prone to further expansions in somatic cells. Tissue specificity of somatic instability was likely influenced by types and structure of repeat interruptions, and additionally, by factors clustering within examined families.

### Repeat Interruptions Are Individual Specific Factors Contributing to Lower Level of Somatic Instability of *DMPK* Expansions

**FIGURE 3:**
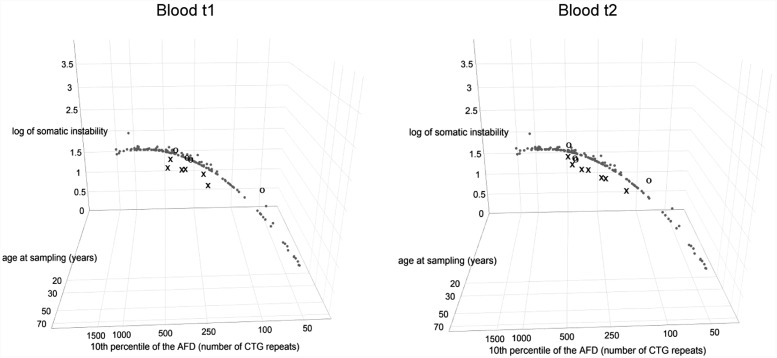
DM1 patients with repeat interruptions show a lower level of somatic instability. The graphs represent the correlation of the level of somatic instability (SI), the 10th percentile (10^th^p) allele length (as an estimate of the progenitor allele length (Higham, 2013)), and age at sampling (AS) according to model 8^∗^. The analyzed group of DM1 patients included our patients with interrupted expansions, our patients with pure expansions (the control group), and 136 patients from the reference group (Morales et al., 2012). Observed level of SI in blood was estimated as the range between the 10th and the 90th percentile of the allele frequency distribution (Morales et al., 2012). Patients with interrupted expansions are shown in X and the control group is shown in O, at the first time point (blood t1) and the second time point (blood t2) (2.5–4 years later for patients with interrupted expansions and 4–11 years later for the control group). Patients with interrupted *DMPK* expansions showed a large residual variance, and the median of their standardized residuals of SI was significantly lower relative to the reference group. The control group showed no difference. AFD, allele frequency distribution.

**FIGURE 4:**
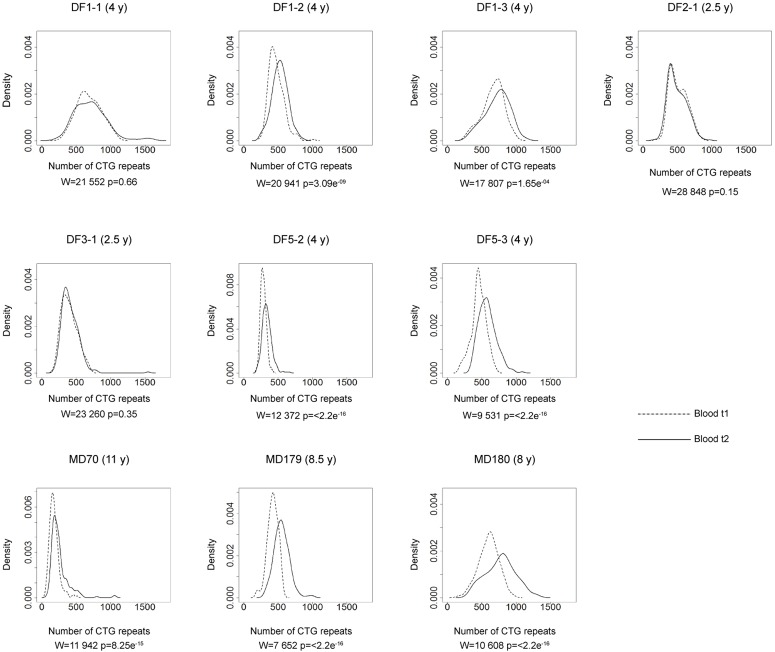
Somatic instability of *DMPK* expansions in blood cells over time. Density plots show the allele frequency distributions from blood samples at the first time point (t1, dashed lines) and the second time point (t2, solid lines). All allele frequency distributions were derived from sizing at least 200 alleles and their comparisons were performed by Wilcoxon–Mann–Whitney test (*W* and *p*-values are shown). Among patients with repeat interruptions, DF1-1, DF2-1, and DF3-1 showed no difference in the allele frequency distribution during the examined time interval, while DF1-2, DF1-3, DF3-2, and DF5-2 showed statistical difference. As expected, patients with uninterrupted repeats (DF5-3 – sister of DF5-2, MD70, MD179 and MD180), showed statistical difference. Time interval in years (y) between sampling at t1 and t2 is indicated in parenthesis, beside patient’s ID.

All patients with interrupted *DMPK* expansions had lower level of observed somatic instability than expected based on the progenitor allele length and age at sampling (Figure 3 and Supplementary Table 5) and the difference was statistically significant (*V* = 0, *p* = 0.03 for the first time point; *V* = 0, *p* = 0.016 for the second time point). In line with no statistical difference between the observed and expected levels of somatic instability in the reference group (*V* = 5132, *p* = 0.18), the control group also showed no difference (*V* = 7, *p* = 0.625 for both time points) (Figure 3 and Supplementary Table 5). It is of note that the patient with a single *de novo* CTC interruption (DF5-2) was characterized by a lower level of somatic instability at both time points in comparison to her sister (DF5-3) with similar expansion size and age at sampling, but without the interruption (Figure 4 and Supplementary Table 5). These results indicated that interrupted *DMPK* expansions were more stable relative to expansions with pure CTG repeats in blood cells and this feature was maintained over time.

Patients with interrupted *DMPK* expansions showed a noticeably larger residual variance of somatic instability according to model 8^∗^, and median of standardized residuals was statistically lower in patients with interrupted *DMPK* expansions relative to the reference group (*W* = 727, *p* = 1.2e^-3^ for the first time point and *W* = 864, *p* = 2.8e^-4^ for the second time point) (Figure 3). On the contrary, median of standardized residuals of somatic instability in the control group showed no statistical difference relative to the reference group (*W* = 221, *p* = 0.53 for the first time point and *W* = 253, *p* = 0.82 for the second time point) (Figure 3). Since the repeat interruptions were a common factor for patients showing larger individual-specific differences in the level of somatic instability (with negative standardized residuals), these results indicated that repeat interruptions could be individual-specific factors significantly contributing to reduced somatic instability in DM1 patients.

### Interrupted *DMPK* Expansions Show Slower Progression of Somatic Instability Over Time

The comparison of the allele frequency distributions from two time points during the interval of two and a half to four years in DF1-1, DF2-1 and DF3-1 (Figure 4) showed no statistical change (Table 2), indicating a relative stability of interrupted *DMPK* expansions over time. On the other hand, the observed shift in the distribution toward larger allele lengths in DF1-2, DF1-3 and DF5-2 (Figure 4) was statistically significant (Table 2), indicating that the level of somatic instability was still enough to cause a shift of allele frequency distribution toward larger expansions. As expected, the control group had statistically significant shift in the distributions toward larger allele lengths during time interval of 4–11 years (Figure 4 and Table 2).

**Table 2:** Comparisons of the allele frequency distributions of patients with interrupted and uninterrupted *DMPK* expansions obtained from blood samples at two time points, and the observed and expected expansion size increments over time.

Patient ID	Mode t1	Mode t2	Mode (5′ end) t1	Mode (5′ end) t2	Wilcoxon–Mann–Whitney test		Time between two samplings (years)	Observed increment	Expected increment	Expected increment (5′ end)	Wilcoxon signed-rank test	
										
					*W*	*P*-value					*V*	*P* value
DF1-1	612	720	577	685	21,552	0.66	4	108	147	142	0	**0.02**
DF1-2	417	527	382	492	20,941	**3.09e^-09^**	4	110	119	114		
DF1-3	732	779	697	744	17,807	**1.65e^-04^**	4	47	165	160		
DF2-1	410	401	325	316	28,848	0.15	2.5	-9	62	50		
DF3-1	335	345	296	306	23,260	0.35	2.5	10	51	46		
DF5-2	267	324	238	295	12,372	**<2.2e^-16^**	4	57	97	93		
DF5-3^∗^	445	565	445	565	9,531	**<2.2e^-16^**	4	120	123	123	0	0.125
MD70^∗^	158	186	158	186	11,492	**8.25e^-15^**	11	28	340	340		
MD179^∗^	427	540	427	540	7,652	**<2.2e^-16^**	8.5	113	287	287		
MD180^∗^	622	806	622	806	10,608	**<2.2e^-16^**	8	184	297	297		


*Mode, the mode allele length in the number of repeats; t1 and t2, the first and the second time point, respectively; W, statistics of the Wilcoxon–Mann–Whitney test used for comparison of allele frequency distribution at two time points in each patient; The observed expansion size increment over time was calculated as a difference between the mode allele length at the first and the second time point both for the entire interrupted *DMPK* expansions and their 5′ ends and had the same values. The expected expansion size increment over time was calculated using the equation of a linear regression model for uninterrupted *DMPK* expansions that shows a correlation between expansion size increment, initial expansion in the number of repeats (IE) and time in years between sampling (T): increment = -90 + 37xT + 0.146xIE (Martorell et al., 1998). As a measure of IE we used the mode allele length inferred from more than 200 sized alleles in each sample. V, statistics of the Wilcoxon signed-rank test used for comparison of the observed and expected expansion size increment over time, as well as the observed and expected expansion size increment at the 5′ end over time in each group of patients (the same values were obtained). ^∗^Analyzed patients with uninterrupted *DMPK* expansions.*

The observed expansion size increment over time was statistically lower than expected based on the mode allele length and time interval between samplings only in patients with interrupted *DMPK* expansions (*V* = 0, *p* = 0.03), but not in the control group (*V* = 0, *p* = 0.13) (Table 2). Both sisters from the DF5 family were characterized by a shift in the distribution toward larger allele lengths during the same time interval of 4 years. However, DF5-2 with a single *de novo* CTC interruption, showed the smaller expansion size increment over time relative to DF5-3 with similar expansion size, but without interruptions (Figure 4). These results indicated that patients with interrupted *DMPK* expansions were characterized by a slower progression of somatic expansions in comparison to patients with uninterrupted expansions in blood cells.

### Repeat Interruptions Are Stable in Somatic Cells and *in Cis* Stabilize the Remaining Part of *DMPK* Expansions

To examine the influence of repeat interruptions on the demonstrated lower level and slower progression of somatic instability at the *DMPK* locus, we separately characterized mutational dynamics of the 3′ end, containing repeat interruptions, and the 5′ end, the remaining part of expansions.

**FIGURE 5:**
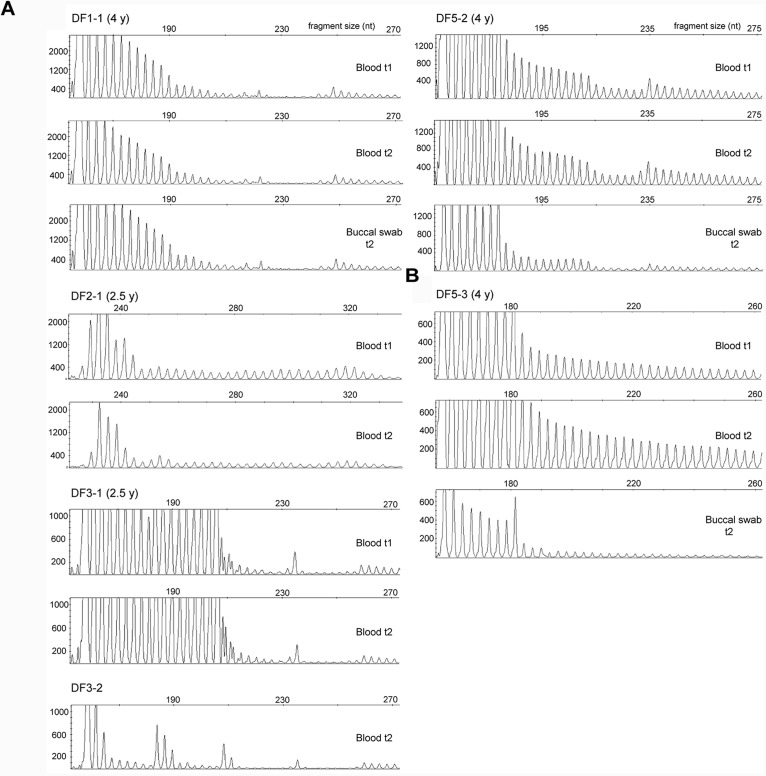
Somatic stability of repeat interruptions at the 3′ end of *DMPK* expansions between tissues and over time. **(A)** Identical repeat-primed PCR profiles in blood sample at the first (t1) and the second time point (t2) and in buccal swab sample (only for patients DF1-1 and DF5-2) in patients with interrupted *DMPK* expansions. All profiles were obtained using repeat-binding primer P4CTG-for (Pešović et al., 2017). The only exception was DF2-1, whose profiles were obtained using P5CCG primer, binding to a large CCG block containing 36 repeats. DF1-2 and DF1-3 are not shown, as they had the identical repeat-primed PCR profiles from all analyzed samples as their mother DF1-1 due to identical wild-type allele of 5 CTG repeats and the same pattern of repeat interruptions. **(B)** Repeat-primed PCR profiles in blood sample at the first time point (t1) and the second time point (t2) and in buccal swab sample in patient DF5-3 with an uninterrupted *DMPK* expansion. The profiles were obtained using repeat-binding primer P4CTG-for. Time interval in years (y) between sampling at t1 and t2 is indicated in parenthesis, beside patient’s ID.

For each examined patient, repeat-primed PCR profiles of the 3′ end were identical in buccal and blood cells, and in blood cells sampled at two time points (Figure 5). Blood cells at two time points and buccal cells were characterized by identical structure of CCGCTG hexamer runs in DF1 family, and by a single CTC repeat in DF5-2. Identical structures of small CCG blocks were identified in blood cells of DF3-1 over time. Additional characterization of repeat interruptions in DF2-1 showed the presence of previously assumed large CCG block containing 36 repeats, similar to his daughter (DF2-2) who was characterized by a block of 40 CCG repeats (Pešović et al., 2017). This interruption also showed the identical structure in blood cells over time. The obtained results demonstrated a relative somatic stability of different types and patterns of repeat interruptions at the 3′ end of *DMPK* expansions in two examined tissues and throughout time in blood cells.

The 5′ ends showed lower level of the observed somatic instability than expected for the corresponding progenitor allele length and age at sampling (*V* = 0, *p* = 0.03 for the first time point; *V* = 0, *p* = 0.04 for the second time point) (Supplementary Table 6). No statistical difference was observed neither in the control group, nor the reference group according to the retested model (data not shown). In line with the results for the whole run of repeats in interrupted *DMPK* expansions, the comparison of the 5′ end frequency distributions at two time points showed no statistical change in DF1-1, DF2-1, and DF3-1, and a statistically significant shift toward larger allele lengths in DF1-2, DF1-3, and DF5-2 (Table 2). The 5′ ends also showed statistically smaller observed increment than expected based on the mode allele length and time interval between samplings (*V* = 0, *p* = 0.04, Table 2). A lower level and a slower progression of somatic instability at the 5′ end of interrupted *DMPK* expansions were suggestive of a stabilizing effect of repeat interruptions not only at the 3′ end, but also on the remaining part of *DMPK* expansions. Collectively, these results established repeat interruptions as *cis*-acting factors with a stabilizing effect at the *DMPK* locus in blood cells.

### Repeat Interruptions Are Positive Genetic Modifiers of Age at Onset in DM1 Patients

According to model 10^∗^ (correlating age at onset with the progenitor allele length), the observed age at onset was 11–23 years higher than expected in four out of seven patients with repeat interruptions (DF1-1, DF1-2, DF2-1, and DF3-1) (Supplementary Table 7). Model 11^∗^ (correlating age at onset with the progenitor allele length and residual variance of somatic instability obtained from model 8^∗^) gave a somewhat better prediction, but the observed age at onset was still higher than expected (7–20 years) in the above mentioned patients (Supplementary Table 7). Both models closely predicted age at onset in the control group (Supplementary Table 7). However, there was no statistical difference between the observed and expected age at onset neither in the group of patients with repeat interruptions, nor in the control group according to models 10^∗^ and 11^∗^ (Supplementary Table 7).

**FIGURE 6:**
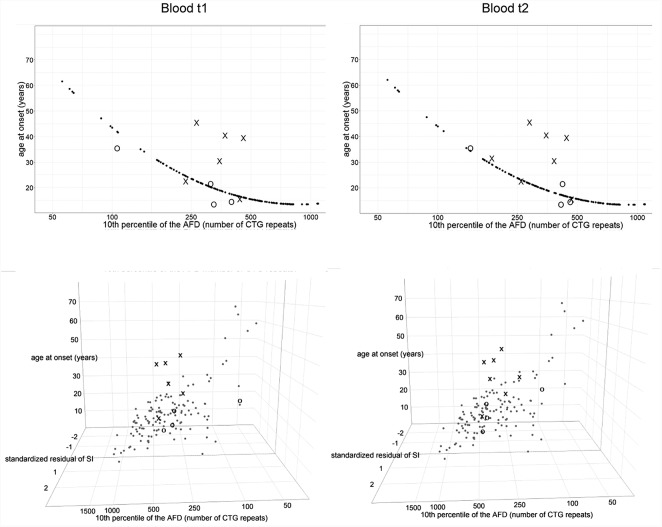
Repeat interruptions are positive genetic modifiers of age at onset in DM1 patients. The graphs represent the correlation of age at onset (AO) with the 10th percentile (10^th^p) allele length (as an estimate of the progenitor allele length, Higham, 2013) according to model 10^∗^**(top graphs)**, and additionally with standardized residual of somatic instability (SI) according to model 11^∗^ **(bottom graphs)**. The analyzed group of DM1 patients included our patients with interrupted expansions, our patients with pure expansions (the control group), and 121 symptomatic patients from the reference group (Morales et al., 2012). Observed values of AO (self-reported by patients) are shown in X in patients with interrupted expansions and in O in the control group, at the first time point (blood t1) and the second time point (blood t2) (2.5–4 years later for patients with interrupted expansions and 4–11 years later for the control group). Patients with interrupted expansions showed a large residual variance when AO was correlated with the 10th allele length (model 10^∗^) and median of their standardized residuals was statistically higher than in the reference group **(top graphs)**. The inclusion of residual variance of SI as a variable (model 11^∗^) resulted in no statistical difference in medians of standardized residuals between patients with interrupted expansions and the reference group **(bottom graphs)**. The control group showed no statistical difference according to both models. AFD, allele frequency distribution.

We considered residual variance in models 10^∗^ and 11^∗^ to examine inter-individual variability of age at onset. According to model 10^∗^, patients with interrupted *DMPK* expansions showed a noticeably larger residual variance, and median of their standardized residuals was statistically higher than in the reference group (*W* = 147, *p* = 0.01 for the first time point and *W* = 171, *p* = 0.008 for the second time point) (Figure 6). The control group showed no statistical difference (*W* = 289, *p* = 0.51 for the first time point and *W* = 218, *p* = 0.74 for the second time point) (Figure 6). This result indicated the existence of factors with a significant contribution to individual-specific differences in age at onset in patients carrying repeat interruptions. Notably, inclusion of residual variance of somatic instability (according to model 8^∗^) as a variable in model 11^∗^, resulted in no statistical difference in medians of standardized residuals between patients with interrupted expansions and the reference group (*W* = 207, *p* = 0.08 for the first time point and *W* = 252, *p* = 0.07 for the second time point) (Figure 6). The control group again showed no statistical difference (*W* = 249, *p* = 0.93 for the first time point and *W* = 204, *p* = 0.6 for the second time point) (Figure 6). This result indicated that individual-specific differences in the level of somatic instability could explain individual-specific differences in age at onset, observed in patients carrying repeat interruptions according to model 10^∗^. In addition, median of standardized residuals in age at onset in model 11^∗^ was statistically lower than in model 10^∗^ in patients with interrupted *DMPK* expansions (*V* = 0, *p* = 0.03 for the first time point and *V* = 0, *p* = 0.02 for the second time point), but not in the control group (*V* = 6, *p* = 0.88 for the first time point and *V* = 6, *p* = 0.88 for the second time point), nor the reference group (*V* = 3854, *p* = 0.67). According to this result, individual specific differences in the level of somatic instability had a greater impact on age at onset in patients with interrupted expansions. Given that repeat interruptions significantly contributed to individual-specific differences in the level of somatic instability by stabilization of *DMPK* expansions in somatic cells, these results implied that repeat interruptions could be considered as factors with a positive modifying effect on age at onset in DM1 patients.

## Discussion

This study reports on the characterization of mutational dynamics of *DMPK* expanded alleles in somatic cells in DM1 patients carrying repeat interruptions and how this dynamics influences the age at onset. The novelty of our study is the finding that repeat interruptions contribute to later appearance of disease symptoms and the mechanism by which they operate is a genetic stabilization of *DMPK* expansions in somatic cells. In addition, we showed that mutational dynamics of interrupted *DMPK* expansions was tissue-specific and the structures of repeat interruptions were stable in somatic tissues over time.

We used single-molecule small-pool PCR to quantify somatic instability at the *DMPK* locus in our previously characterized DM1 patients with repeat interruptions (Pešović et al., 2017). In that study, the progenitor allele length was estimated as a sharp lower boundary of expansion range (Monckton et al., 1995), since we used small-pool PCR with ∼300 pg of input DNA. This kind of estimation of the progenitor allele length was not clear and could have been biased when our patients were analyzed by single-molecule small pool PCR. Instead, we used the 10th percentile allele length as an unbiased measurement (Higham, 2013) and obtained approximately the same length of the progenitor alleles. All retested linear regression models using the 10th percentile allele length explained almost the same percent of variability of somatic instability and age at onset as the original models (Morales et al., 2012), additionally demonstrating that the 10th percentile allele length was a precise measurement of the progenitor allele length. This enabled us to use the originally studied large group of patients (Morales et al., 2012) as a valuable reference group for comparisons of somatic instability and age at onset. Since the limited number of DM1 patients was available for our study, quantification of somatic instability at two sampling time points, followed by the same results in statistical analyses, supported the reliability of our results. Testing of the control DM1 patients, who lacked repeat interruptions and were not matched by expansion size and age at sampling, showed a proper fit to the applied liner regression models (Martorell et al., 1998; Morales et al., 2012). Analysis of the control group additionally confirmed the reliability of the obtained results and showed the absence of possible population-specific differences relative to the reference group. Therefore, our approach to study somatic instability and age at onset in DM1 patients with repeat interruptions does not require a group of patients with uninterrupted expansions matched by expansion size and age at sampling. As such, it may offer an advantage for future studies that would enroll a larger number of patients from different countries (Wood et al., 2018).

The similar approach to study somatic instability of interrupted expansions in DM1 patients was used by Braida et al. (2010) with only difference being the estimate of the progenitor allele size as originally proposed (Monckton et al., 1995). Two recently published studies compared small-pool PCR profiles (Tomé et al., 2018) and a degree of somatic instability, measured as a difference between the progenitor and modal allele length (Cumming et al., 2018), in patients with interruptions relative to a few age matched patients with uninterrupted expansions of similar size. However, comparisons to a large group of DM1 patients with uninterrupted expansions better address variability in somatic instability seen in DM1.

Observed allele frequency distributions that are skewed toward larger allele lengths and characterized by differences between two time points in blood cells, as well as between blood and buccal cells in each patient, indicate that somatic mosaicism of interrupted *DMPK* expansions has characteristics similar to expansions consisting of pure (CTG)_n_ tract: a bias toward further expansions throughout the patient’s life, an age related dependency and a tissue specificity (Monckton et al., 1995; Wong et al., 1995; Martorell et al., 1998). However, there is a principal difference in their mutational dynamics, because the interrupted *DMPK* expansions are more stable. In particular, we demonstrated that patients with interrupted *DMPK* expansions had different mutational dynamics in blood and buccal cells that was likely influenced by types and structure of repeat interruptions, and additionally by factors clustering within examined families. Patients with interrupted *DMPK* expansions were characterized by a lower level of allele size heterogeneity in blood cells than expected for the progenitor allele length and age at sampling. They also showed larger individual-specific differences in the level of somatic instability, which additionally confirmed reduced level of somatic instability. These individual-specific differences can be attributed to repeat interruptions due to their stabilizing effect on *DMPK* expansions (as discussed below). The similar result was reported for members of the family with *DMPK* expansions with multiple GGC and CCG interruptions at the 3′ end relative to the same reference group (Braida et al., 2010), although the effect of other factors clustering within this family and potentially influencing variability of somatic instability (Morales et al., 2012) cannot be excluded. Factors contributing to individual-specific differences in the level of somatic instability are mostly unknown. Our results demonstrate that repeat interruptions are the individual-specific, genetic modifier of somatic instability. Among other genetic factors, the single nucleotide variant in *MSH3* mismatch repair gene is the only known *trans*-acting modifier of somatic instability in DM1 patients (Morales et al., 2016). We further showed that patients with interrupted *DMPK* expansions were characterized by the lower expansion size increment in blood cells over time than expected for the corresponding allele length and time interval between two samplings. Three examined patients were even characterized by a relative stability of interrupted *DMPK* expansions over time. Patient with a single *de novo* CTC interruption (DF5-2) showed the smaller expansion size increment over the same time interval relative to her sister with similar expansion size, but without interruptions (DF5-3). The smaller net expansion gain over time in our DM1 patients with repeat interruptions at the 3′ end is in line with the finding in two DM1 patients with repeat interruptions at the 5′ end (Tomé et al., 2018).

Our findings extend the previous knowledge of mutational dynamics of interrupted *DMPK* expansions, and collectively with previous studies (Braida et al., 2010; Cumming et al., 2018; Tomé et al., 2018), indicate that different types and structures of repeat interruptions at both ends of expansions, including only a single interruption and *de novo* arisen interruptions, confer genetic stability at the *DMPK* locus in somatic cells. Repeat interruptions were also identified in expanded alleles associated with SCA8 (Moseley et al., 2000; Hu et al., 2017), SCA10 (Matsuura et al., 2006; Landrian et al., 2017) and SCA17 (Gao et al., 2008), but their influence on somatic instability was studied only in SCA17, showing a greater stability of *TBP* expansions interrupted by CAA repeats (Gao et al., 2008).

The greater stability of interrupted *DMPK* expansions in somatic cells is a consequence of *in cis* stabilizing effect of repeat interruptions, both locally and beyond the part of expansion harboring interruptions. We found the identical structures of repeat interruptions at the 3′ end of *DMPK* expansions in blood and buccal cells, and in blood cells over time. This feature of somatic tissues is in contrast to germline tissues, since intergenerational changes in the structures of repeat interruptions were reported (our DF2 family and two families reported by Musova et al., 2009). Although repeat interruptions do not completely abolish instability at the *DMPK* locus in somatic cells, we showed that the remaining part (5′ end) of interrupted expansions was more stable than expected for the corresponding allele size and age at sampling. This was previously described for *DMPK* expansions with multiple GGC and CCG interruptions (Braida et al., 2010), and according to our findings it seems to be a general characteristic of *DMPK* expansions with various types and patterns of repeat interruptions, at least at the 3′ end. In addition, we demonstrated that this stability is maintained over time in blood cells, since the 5′ end of interrupted expansions was characterized by lower expansion size increment over time than expected based on the allele length and time interval between two samplings.

The ability of repeat interruptions to interfere with the adoption of mutagenic intermediates by CTG/CAG repeats (slipped DNAs and R-loop) (Pearson et al., 2002; Axford et al., 2013; Reddy et al., 2014) is a likely mechanism that maintains the stable local structure of the part of *DMPK* expansion harboring interruptions. In addition, the presence of repeat interruptions in hairpins may facilitate their correct repair upon recognizing the mismatches that arise from the misalignment of repeat interruptions, at least in yeast cells (Rolfsmeier et al., 2000). The mechanisms that contribute to increased stability of the remaining part of expansions are not known and may involve epigenetic mechanisms. It can be speculated that repeat interruptions may alter nucleosome positioning, since CTG repeats at the *DMPK* locus act as strong nucleosome positioning elements (Wang et al., 1994), and CAT repeat interruptions in SCA1 expanded repeats significantly reduce their ability to assemble into nucleosomes (Mulvihill et al., 2005). In addition, CCG/GGC repeats, the most commonly observed interruptions at *DMPK* expanded alleles, preferentially exclude nucleosome formation (Wang et al., 1996; Mulvihill et al., 2005). Altered nucleosome positioning may modulate the fidelity of DNA metabolic processes involved in repeat instability (Cleary and Pearson, 2003), or may interfere with the action of *cis* elements that are placed in the flanking sequences and confer instability at the *DMPK* locus (Gourdon et al., 1997; Brock et al., 1999). Polarized hypermethylation of CpG islands downstream of the CTG repeats, very rarely observed in juvenile and adult DM1 patients (Barbé et al., 2017), was described in patients with CCG interruptions at the 3′ end of *DMPK* expansion (Santoro et al., 2015). Interestingly, in *E. coli*-based *in vivo* methylation system, CpG methylation of flanking sequences in DM1 patients-derived clones has shown a mild stabilizing effect on expanded CTG repeats (Nichol and Pearson, 2002). However, hypermethylation of flanking CpG islands has not been observed in two DM1 families carrying repeat interruption at the 5′ end (Tomé et al., 2018), suggesting that DNA methylation could not be the main mechanism, or it can merely be a consequence of the presence of CCG interruptions at the 3′ end. Furthermore, it is noteworthy that bioinformatic predictions showed similar secondary structures of RNA formed by various patterns of CCG and CTG repeats without effect on the affinity for MBNL binding (Braida et al., 2010). This finding was supported by the study from Santoro et al. (2013), which demonstrated the presence of nuclear foci in muscle biopsies from DM1 patients with variant repeats and their co-localization with MBNL proteins. Additionally, they confirmed the aberrant splicing events that had been postulated as main pathomechanism underlying DM1. Nevertheless, the interruption patterns described in our patients are unique and additional studies are required to examine the possible effect on formation of nuclear foci and the consequence for RNA metabolism.

It has been assumed that the presentation of DM1 symptoms appears when a sufficient proportion of cells acquire sufficiently long expansions that cause dysfunction, seen at the tissue level (Morales et al., 2012). Similar assumption, known as the somatic threshold model, has been proposed for Huntington’s disease (HD) (Budworth et al., 2015). Indeed, patient cohort studies have shown that individual-specific differences in the level of somatic instability of *DMPK* and *HTT* expansions contribute to variability in age at onset (Swami et al., 2009; Morales et al., 2012). Herein, we demonstrated that individual-specific differences in the level of somatic instability had a greater contribution to age at onset in DM1 patients with repeat interruptions relative to patients with uninterrupted expansions. Since repeat interruptions significantly contribute to individual-specific differences in the level of somatic instability and stabilize *DMPK* expansions in somatic cells, our results indicate the role of repeat interruptions as factors with a positive modifying effect on age at onset in DM1 patients. In general, DM1 patients carrying repeat interruptions have predisposition to develop the disease later than average due to a slower progression of expansions toward larger size than average. Therefore, the stabilizing effect of repeat interruptions at the *DMPK* locus in somatic cells can explain an apparently later age at onset in four out of seven patients in our study. The association of repeat interruptions with later age at onset in DM1 patients has also been reported in other studies (Musova et al., 2009; Botta et al., 2017), as well as for SCA8 patients with interrupted *ATXN8OS* expansions (Hu et al., 2017).

The lack of difference between the observed and expected age at onset in the whole group of analyzed DM1 patients with repeat interruptions likely reflects the influence of additional factors that contribute to variability of age at onset, since the applied linear regression models (Morales et al., 2012) explained up to ∼55% of variability. Contribution of additional factors that influence the individual-specific differences in the age at onset may also explain why intergenerational instability, biased toward contractions in our DF1, DF2, and DF3 families, and in two families described by Botta et al. (2017), was still accompanied by the genetic anticipation (documented as the decrease in age at onset).

Our evidence that repeat interruptions can positively modulate age at onset in DM1 patients, through stabilizing effect on *DMPK* expansions in somatic cells, is in line with the results showing that suppression of somatic instability of CAG expansions substantially delayed the onset of symptoms in HD mouse models with deletion of certain repair genes (*Ogg1, Msh2* and *Msh3*) (Wheeler et al., 2003; Dragileva et al., 2009; Budworth et al., 2015). Additionally, in HD and other patients with polyglutamine diseases, single nucleotide variants in DNA repair genes have been identified as genetic factors that modify age at onset, probably by influencing somatic instability (Genetic Modifiers of Huntington’s Disease (GeM-HD) Consortium, 2015; Bettencourt et al., 2016). Importantly, a study on the HD mouse model has shown that pharmacological treatment that suppresses somatic expansions during life rescues motor decline in these animals (Budworth et al., 2015). Our results on age at onset in DM1 patients with repeat interruptions and the reduced level of somatic instability point to the significance of somatic instability for the disease course and support studies on pharmacological agents that suppress somatic expansions with a potential to delay disease progression.

By detailed characterization of mutational dynamics of *DMPK* expansions in somatic cells, our study has provided firm evidence that various types and patterns of repeat interruptions, including only a single *de novo* interruption, *in cis* confer stability to *DMPK* expansions in somatic cells. Due to stabilizing effect on the *DMPK* locus in somatic cells, repeat interruptions predispose DM1 patients to develop disease later than average, supporting the assumption that they can act as genetic modifier of DM1 phenotype. Our results underlie the clinical significance of repeat interruptions and somatic instability of *DMPK* expansions for progression of the disease. Studies on larger groups of DM1 patients are needed to further elucidate phenotypic consequences of repeat interruptions. They would be important for appropriate genetic counseling, improved stratification of patients in clinical trials and new potential therapeutic approaches.

## Author Contributions

JP performed genetic and statistical analysis and drafted the manuscript. SP examined patients, collected the samples, and provided clinical data. MB verified statistical analysis. GB supported genetic analyses. VR-S examined patients and reviewed the manuscript. DS-P designed the study and reviewed the manuscript. All authors read and approved the final manuscript.

## Conflict of Interest Statement

The authors declare that the research was conducted in the absence of any commercial or financial relationships that could be construed as a potential conflict of interest.
