# Supplementary material for: Repeat Interruptions Modify Age at Onset in Myotonic Dystrophy Type 1 by Stabilizing DMPK Expansions in Somatic Cells
Source: Front Genet. 2018 Nov 27;9:601. doi: 10.3389/fgene.2018.00601 (PMC6278776; doi:10.3389/fgene.2018.00601)
Supplement: Supplementary file 1 [file Data_Sheet_1.pdf]

Supplementary Material

Repeat interruptions modify age at onset in myotonic dystrophy type 1 by stabilizing *DMPK* expansions in somatic cells

Jovan Pešović[1*], Stojan Perić[2,3], Miloš Brkušanin[1], Goran Brajušković[1], Vidosava Rakočević-Stojanović[2,3] & Dušanka Savić-Pavićević[1*]

[1]Center for Human Molecular Genetics, Faculty of Biology, University of Belgrade, Belgrade, Serbia
[2]School of Medicine, University of Belgrade, Belgrade, Serbia
[3]Neurology Clinic, Clinical Center of Serbia, Belgrade, Serbia

Correspondence:
Prof. Dušanka Savić-Pavićević, E-mail address: duska@bio.bg.ac.rs; ORCID ID: 0000-0002-2079-4077
Jovan Pešović, E-mail address: jovan.pesovic@bio.bg.ac.rs; ORCID ID: 0000-0002-8304-2067
Center for Human Molecular Genetics, Faculty of Biology, University of Belgrade, Belgrade, Serbia

Supplementary Table 1. **Linear regression models of correlation between level of somatic instability (SI), the 10th percentile allele length (10thp) and age at sampling (AS) in DM1 patients including the reference group of 136 patients with pure *DMPK* expansions (Morales et al., 2012) and our patients observed at the first time point (six with interrupted and four with pure *DMPK* expansions) and the second time point (seven with interrupted and four with pure *DMPK* expansions).**

Model	Term	Coefficient	Standard error	t-statistics	Pr(>|t|)	R^2 (adjusted)	P value
**The first time point**							
Model 4*: $\log(SI) = \beta_0 + \beta_1\log_{10}(10^{th}p)$	Intercept	β_0 = -1.0027	0.1916	-5.234	$5.75e^{-07}$	0.69	**$<2.2e^{-16}$**
	$\log_{10}(10^{th}p)$	β_1 = 1.3500	0.0754	17.905	$<2e^{-16}$		
Model 6*: $\log(SI) = \beta_0 + \beta_1\log_{10}(10^{th}p) + \beta_2\log_{10}(AS)$	Intercept	β_0 = -2.16990	0.24612	-8.817	$3.67e^{-15}$	0.76	**$<2.2e^{-16}$**
	$\log_{10}(10^{th}p)$	β_1 = 1.46048	0.06857	21.300	$<2e^{-16}$		
	$\log_{10}(AS)$	β_2= 0.59450	0.09123	6.517	$1.15e^{-09}$		
Model 7*: $\log(SI) = \beta_0 + \beta_1\log_{10}(10^{th}p) + \beta_2\log_{10}(AS) + \beta_3\log_{10}(10^{th}p)x\log_{10}(AS)$	Intercept	β_0= 0.2295	0.7644	0.300	0.76	0.77	**$<2.2e^{-16}$**
	$\log_{10}(10^{th}p)$	β_1 = 0.5147	0.2939	1.751	0.08		
	$\log_{10}(AS)$	β_2= -0.9658	0.4806	-2.010	0.05		
	$\log_{10}(10^{th}p)x\log_{10}(AS)$	β_3= 0.6181	0.1871	3.303	0.001		
Model 8*: $\log(SI) = \beta_0 + \beta_1\log_{10}(10^{th}p) + \beta_2\log_{10}(AS) + \beta_3\log_{10}(10^{th}p)x\log_{10}(AS) + \beta_4\log_{10}(10^{th}p)^2 + \beta_5\log_{10}(AS)^2$	Intercept	β_0= -9.2774	0.9226	-10.056	$<2e^{-16}$	0.90	**$<2.2e^{-16}$**
	$\log_{10}(10^{th}p)$	β_1 = 8.7331	0.6442	13.556	$<2e^{-16}$		
	$\log_{10}(AS)$	β_2= -1.3453	0.5828	-2.308	0.02		
	$\log_{10}(10^{th}p)x\log_{10}(AS)$	β_3= 0.3962	0.1393	2.844	0.005		
	$\log_{10}(10^{th}p)^2$	β_4= -1.6443	0.1242	-13.237	$<2e^{-16}$		
	$\log_{10}(AS)^2$	β_5= 0.3522	0.1343	2.622	0.01		

Supplementary Table 1. C**ontinued**

Model	Term	Coefficient	Standard error	t-statistics	Pr(>|t|)	R^2 (adjusted)	P value
**The second time point**							
Model 4*: $\log(SI) = \beta_0 + \beta_1\log_{10}(10^{th}p)$	Intercept	$\beta_0 = -1.01328$	0.19178	-5.284	$4.55e^{-07}$	0.69	**$<2.2e^{-16}$**
	$\log_{10}(10^{th}p)$	$\beta_1 = 1.35478$	0.07543	17.961	$<2e^{-16}$		
Model 6*: $\log(SI) = \beta_0 + \beta_1\log_{10}(10^{th}p) + \beta_2\log_{10}(AS)$	Intercept	$\beta_0 = -2.21356$	0.24576	-9.007	$1.16e^{-15}$	0.76	**$<2.2e^{-16}$**
	$\log_{10}(10^{th}p)$	$\beta_1 = 1.46867$	0.06826	21.517	$<2e^{-16}$		
	$\log_{10}(AS)$	$\beta_2 = 0.60874$	0.09094	6.694	$4.51e^{-10}$		
Model 7*: $\log(SI) = \beta_0 + \beta_1\log_{10}(10^{th}p) + \beta_2\log_{10}(AS) + \beta_3\log_{10}(10^{th}p)x\log_{10}(AS)$	Intercept	$\beta_0 = 0.1829$	0.7600	0.241	0.81	0.78	**$<2.2e^{-16}$**
	$\log_{10}(10^{th}p)$	$\beta_1 = 0.5245$	0.2920	1.796	0.07		
	$\log_{10}(AS)$	$\beta_2 = -0.9499$	0.4777	-1.989	0.05		
	$\log_{10}(10^{th}p)x\log_{10}(AS)$	$\beta_3 = 0.6171$	0.1859	3.320	0.001		
Model 8*: $\log(SI) = \beta_0 + \beta_1\log_{10}(10^{th}p) + \beta_2\log_{10}(AS) + \beta_3\log_{10}(10^{th}p)x\log_{10}(AS) + \beta_4\log_{10}(10^{th}p)^2 + \beta_5\log_{10}(AS)^2$	Intercept	$\beta_0 = -9.2521$	0.9103	-10.164	$<2e^{-16}$	0.90	**$<2.2e^{-16}$**
	$\log_{10}(10^{th}p)$	$\beta_1 = 8.7348$	0.6340	13.776	$<2e^{-16}$		
	$\log_{10}(AS)$	$\beta_2 = -1.4005$	0.5725	-2.446	0.02		
	$\log_{10}(10^{th}p)x\log_{10}(AS)$	$\beta_3 = 0.4069$	0.1374	2.962	0.004		
	$\log_{10}(10^{th}p)^2$	$\beta_4 = -1.6460$	0.1221	-13.483	$<2e^{-16}$		
	$\log_{10}(AS)^2$	$\beta_5 = 0.3616$	0.1312	2.757	0.007		

The published linear regression models explaining the variability of somatic instability (Morales et al., 2012) were retested using the 10th percentile allele length (as an estimation of the progenitor allele length (Higham, 2013)) and age at sampling as independent variables. All models were slightly improved since values of the adjusted squared coefficient of correlation (adjusted R^2) were higher than the original ones (Morales et al., 2012). P value – statistical significance for each model; Coefficient – an indication of the strength of the effect of each independent variable to the model and its associated standard error; t-statistic and Pr(>|t|) – indications of the statistical significance that an independent variable is contributing to the explanatory power of the model.

Supplementary Table 2. **Linear regression models of correlation between the level of somatic instability (SI) of *DMPK* expansions, the 10th percentile allele length (10^{th}p) and age at sampling (AS) in DM1 patients including the reference group of 136 patients with pure *DMPK* expansions (Morales et al., 2012) and our patients analyzed at both time points (six/seven with interrupted and four with pure *DMPK* expansions). The size of the 5′ ends was considered for the patients with interrupted *DMPK* expansions.**

Model	Term	Coefficient	Standard error	t-statistics	Pr(>|t|)	R^2 (adjusted)	P value
**The first time point**							
Model 8**: $\log(SI) = \beta_0 + \beta_1\log_{10}(10^{th}p) + \beta_2\log_{10}(AS) + \beta_3\log_{10}(10^{th}p)x\log_{10}(AS) + \beta_4\log_{10}(10^{th}p)^2 + \beta_5\log_{10}(AS)^2$	Intercept	β_0= -9.3401	0.9048	-10.323	$<2e^{-16}$	0.90	**$<2.2e^{-16}$**
	$\log_{10}(10^{th}p)$	β_1 = 8.8148	0.6308	13.974	$<2e^{-16}$		
	$\log_{10}(AS)$	β_2= -1.4058	0.5733	-2.452	0.02		
	$\log_{10}(10^{th}p)x\log_{10}(AS)$	β_3= 0.4063	0.1370	2.966	0.004		
	$\log_{10}(10^{th}p)^2$	β_4= -1.6636	0.1216	-13.683	$<2e^{-16}$		
	$\log_{10}(AS)^2$	β_5= 0.3684	0.1320	2.790	0.006		
**The second time point**							
Model 8**: $\log(SI) = \beta_0 + \beta_1\log_{10}(10^{th}p) + \beta_2\log_{10}(AS) + \beta_3\log_{10}(10^{th}p)x\log_{10}(AS) + \beta_4\log_{10}(10^{th}p)^2 + \beta_5\log_{10}(AS)^2$	Intercept	β_0= -9.3412	0.8887	-10.511	$<2e^{-16}$	0.90	**$<2.2e^{-16}$**
	$\log_{10}(10^{th}p)$	β_1 = 8.8493	0.6180	14.319	$<2e^{-16}$		
	$\log_{10}(AS)$	β_2= -1.4704	0.5607	-2.622	0.009		
	$\log_{10}(10^{th}p)x\log_{10}(AS)$	β_3= 0.4197	0.1345	3.120	0.002		
	$\log_{10}(10^{th}p)^2$	β_4= -1.6737	0.1190	-14.068	$<2e^{-16}$		
	$\log_{10}(AS)^2$	β_5= 0.3789	0.1283	2.953	0.003		

The published linear regression model 8 explaining the variability of somatic instability (Morales et al., 2012) was retested using the 10th percentile allele length (as an estimation of the progenitor allele length (Higham, 2013)) and age at sampling as independent variables. The size of the 5′ ends of interrupted *DMPK* expansions were obtained by subtracting the corresponding interrupted parts at the 3′ end from all detected expanded alleles. P value – statistical significance for each model; Coefficient – an indication of the strength of the effect of each independent variable to the model and its associated standard error; t-statistic and Pr(>|t|) – indications of the statistical significance that an independent variable is contributing to the explanatory power of the model.

Supplementary Table 3. **Linear regression models of correlation between the age at onset (AO), the 10th percentile allele length (10thp) and standardized residuals of somatic instability (SI) in DM1 patients including the reference sample of 121 symptomatic patients with uninterrupted *DMPK* expansions (Morales et al., 2012) and our patients observed at the first time point (six with interrupted and four with pure *DMPK* expansions) and at the second time point (seven with interrupted and four with pure *DMPK* expansions).**

Model	Term	Coefficient	Standard error	t-statistics	Pr(>|t|)	R^2 (adjusted)	P value
**The first time point**							
Model 9*: AO = β_0 + $\beta_1\log_{10}(10^{th}p)$	Intercept	β_0 = 105.354	8.552	12.320	<2e-16	0.43	**<2.2e-16**
	$\log_{10}(10^{th}p)$	β_1 = -32.939	3.321	-9.918	<2e-16		
Model 10*: AO = β_0 + $\beta_1\log_{10}(10^{th}p)$ + $\beta_2\log_{10}(10^{th}p)^2$	Intercept	β_0 = 304.963	50.612	6.026	1.67e-08	0.49	**<2.2e-16**
	$\log_{10}(10^{th}p)$	β_1 = -197.944	41.419	-4.779	4.75e-06		
	$\log_{10}(10^{th}p)^2$	β_2= 33.605	8.411	3.995	1.08e-04		
Model 11*: AO = β_0 + $\beta_1\log_{10}(10^{th}p)$ + $\beta_2$standardized residual of SI + $\beta_3\log_{10}(10^{th}p)^2$	Intercept	β_0 = 304.294	47.696	6.380	3.02e-09	0.55	**<2.2e-16**
	$\log_{10}(10^{th}p)$	β_1 = -197.576	39.032	-5.062	1.42e-06		
	standardized residual(SI)	β_2 = 33.565	0.813	-4.139	6.31e-05		
	$\log_{10}(10^{th}p)^2$	B_3 = -3.365	7.926	4.235	4.36e-05		
**The second time point**							
Model 9*: AO = β_0 + $\beta_1\log_{10}(10^{th}p)$	Intercept	β_0 = 106.435	8.528	12.48	<2e-16	0.43	**<2.2e-16**
	$\log_{10}(10^{th}p)$	β_1 = -33.304	3.310	-10.06	<2e-16		
Model 10*: AO = β_0 + $\beta_1\log_{10}(10^{th}p)$ + $\beta_2\log_{10}(10^{th}p)^2$	Intercept	β_0 = 304.107	50.777	5.989	1.96e-08	0.49	**<2.2e-16**
	$\log_{10}(10^{th}p)$	β_1 = -196.532	41.512	-4.734	5.70e-06		
	$\log_{10}(10^{th}p)^2$	β_2= 33.214	8.423	3.943	1.31e-04		
Model 11*: AO = β_0 + $\beta_1\log_{10}(10^{th}p)$ + $\beta_2$standardized residual of SI + $\beta_3\log_{10}(10^{th}p)^2$	Intercept	β_0 = 303.1017	47.8211	6.338	3.64e-09	0.55	**<2.2e-16**
	$\log_{10}(10^{th}p)$	β_1 = -195.853	39.0958	-5.010	1.77e-06		
	standardized residual(SI)	β_2 = -3.3680	0.8064	-4.177	5.44e-05		
	$\log_{10}(10^{th}p)^2$	B_3 = 33.1042	7.9325	4.173	5.51e-05		

The published linear regression models explaining the variability in age at onset (Morales et al., 2012) were retested using the 10th percentile allele length and standardized residuals of somatic instability according to model 8* (not accounted for by the 10th

percentile allele length and age at sampling) as independent variables. P value – statistical significance for each model; Coefficient – an indication of the strength of the effect of each independent variable to the model and its associated standard error; t-statistic and Pr(>|t|) – indications of the statistical significance that the independent variable is contributing to the explanatory power of the model.

Supplementary Table 4. **Dataset of expanded *DMPK* alleles sized in blood cells at the first (t1) and the second (t2) time point and in buccal cells (B) at the second time point in seven patients with interruptions and four patients without interruptions.**
(This table has been submitted as a separate .xlsx file named Table 4.)
At least 200 individual alleles were sized per sample. The age at sampling (AS) and the age at onset (AO) are indicated for each patient. A value of 0.5 has been added to all AS and AO to allow log transformation of the data. * – analyzed patients with uninterrupted *DMPK* expansions

Supplementary Table 5. **The observed and expected level of somatic instability of interrupted and uninterrupted *DMPK* expansions in blood samples at two time points and the observed level of somatic instability in buccal swab samples.**

Patient ID	Blood t1						Blood t2						Buccal swab t2				
	AS	M	10th	90th	SI	E*	AS	M	10th	90th	SI	E*	AS	M	10th	90th	SI
DF1-1	61.5	670	459	920	461	842	65.5	701	443	979	536	873	65.5	732	581	975	394
DF1-2	34.5	454	348	608	260	407	38.5	527	384	670	286	483	38.5	543	433	678	245
DF1-3	35.5	684	441	840	399	505	39.5	745	469	941	472	571	39.5	786	609	950	341
DF2-1	45.5	492	371	697	326	539	48	472	350	688	338	530	48	NA	NA	NA	NA
DF3-1	50	384	266.5	562.5	296	394	52.5	394	289	560	271	455	52.5	NA	NA	NA	NA
DF3-2	NA	NA	NA	NA	NA	NA	31.5	222	187	285	98	157	31.5	NA	NA	NA	NA
DF5-2	26.5	283	235	337	102	209	30.5	330	262	420	158	265	30.5	326	269	401	132
DF5-3*	23.5	454	314	587	273	282	27.5	568	422	758	336	403	27.5	560	420	701	281
MD70*	50.5	168	106	259	153	58	61.5	219	146	421	275	148	61.5	NA	NA	NA	NA
MD179*	14.5	427	326	522	196	226	23	546	415	688	273	353	23	NA	NA	NA	NA
MD180*	22.5	611	400	785	385	341	30.5	785	462	1062	600	460	30.5	NA	NA	NA	NA

t1 and t2 – the first and the second time point, respectively; AS – age at sampling in years; M, 10th and 90th – median, the 10th percentile and the 90th percentile allele length inferred from more than 200 alleles sized in each sample; SI – observed level of somatic instability assumed as the difference in the number of repeats at the 10th and at the 90th percentile allele length; E* – the expected level of somatic instability according to the linear regression model 8* (Supp. Table 1) which shows a correlation of the level of somatic instability, the 10th percentile allele length and age at sampling; * – analyzed patients with uninterrupted *DMPK* expansions; NA – not available.

Supplementary Table 6. **The observed and expected level of somatic instability of the 5′ ends of interrupted *DMPK* expansions at two time points.**

Patient ID	Blood t1						Blood t2					
	AS	M	10th	90th	SI	E*	AS	M	10th	90th	SI	E*
DF1-1	61.5	635	424	885	461	812	65.5	666	408	944	536	838
DF1-2	34.5	419	313	573	260	368	38.5	492	349	635	286	449
DF1-3	35.5	649	406	805	399	482	39.5	710	434	906	472	551
DF2-1	45.5	407	286	612	326	408	48	387	265	603	338	383
DF3-1	50	345	227.5	523.5	296	314	52.5	355	250	521	271	378
DF3-2	NA	NA	NA	NA	NA	NA	31.5	NA	148	246	98	98
DF5-2	26.5	254	206	308	102	171	30.5	302	233	391	158	227

The size of the 5′ ends were obtained by subtracting the corresponding interrupted parts at the 3′ end from all detected expanded alleles in a sample. t1 and t2 – the first and the second time point, respectively; AS – age at sampling in years; M, 10th and 90th – median, the 10th percentile and the 90th percentile allele length inferred from more than 200 sized alleles in each sample; SI – observed level of somatic instability assumed as the difference in the number of repeats at the 10th and at the 90th percentile allele length; E* – the expected level of somatic instability according to the linear regression model 8** (Supp. Table 2) which shows a correlation of the level of somatic instability, the 10th percentile allele length and age at sampling; NA – not available.

Supplementary Table 7. **Comparisons of observed and expected age at onset in patients with interrupted and uninterrupted *DMPK* expansions according to the analyses at two time points.**

Patient ID	AO	Expected AO							
		Blood t1				Blood t2			
		E(10*)	WSRT	E(11*)	WSRT	E(10*)	WSRT	E(11*)	WSRT
DF1-1	39.5	16.2	V=18 P=0.16	21.9	V=17 P=0.22	16.6	V=25 P=0.08	21.3	V=22 P=0.2
DF1-2	30.5	18.9		23.5		18.0		23.3	
DF1-3	15.5	16.5		18.9		16.1		18.0	
DF2-1	40.5	18.2		23.2		19.1		23.7	
DF3-1	45.5	22.5		25.8		21.6		27.1	
DF3-2	31.5	NA		NA		29.0		34.0	
DF5-2	22.5	24.6		31.7		23.1		28.4	
DF5-3*	21.5	20.2	V=1 P=0.25	20.6	V=3 P=0.63	17.1	V=5 P=1	19.0	V=6 P=0.88
MD70*	35.5	41.9		34.0		34.3		29.5	
MD179*	13.5	19.7		20.9		17.2		19.8	
MD180*	14.5	17.4		16.3		16.2		13.8	

AO – observed age at onset in years as self-reported by patients; t1 and t2 – the first and the second time point, respectively; E(10*) – the expected age at onset according to the linear regression model 10* showing correlation between age at onset and the 10th percentile allele length (Supp. Table 3); E(11*) – the expected age at onset according to the linear regression model 11* showing correlation between age at onset, the 10th percentile of allele length and residual variation in somatic instability according to model 8* (not accounted for by the 10th percentile allele length and age at sampling) (Supp. Table 3); WSRT- Wilcoxon signed-rank test;
V – statistics of the Wilcoxon signed-rank test used for comparison of the observed and expected age at onset in each group of patients. * – analyzed patients with uninterrupted *DMPK* expansions.

References

1. Higham, C.F. (2013). Dynamic DNA and human disease: mathematical modelling and statistical inference for myotonic dystrophy type 1 and Huntington disease. [dissertation thesis]. [Glasgow, UK]: University of Glasgow.
2. Morales, F., Couto, J.M., Higham, C.F., Hogg, G., Cuenca, P., Braida, C., et al. (2012). Somatic instability of the expanded CTG triplet repeat in myotonic dystrophy type 1 is a heritable quantitative trait and modifier of disease severity. *Human Molecular Genetics*, *21*(16), 3558-3567. doi:10.1093/hmg/dds185
